# Role of non-chromosomal birth defects on the risk of developing childhood Hodgkin lymphoma: A Children’s Oncology Group study

**DOI:** 10.1002/pbc.30822

**Published:** 2023-12-25

**Authors:** Erin C. Peckham-Gregory, Lucas Maschietto Boff, Jeremy M. Schraw, Logan G. Spector, Amy M. Linabery, Erik B. Erhardt, Karina B. Ribeiro, Carl E. Allen, Michael E. Scheurer, Philip J. Lupo

**Affiliations:** 1 Department of Pediatrics, Section of Hematology-Oncology, Baylor College of Medicine, One Baylor Plaza, Houston, Texas, USA; 2 Department of Pediatrics, Texas Children’s Cancer and Hematology Centers, Texas Children’s Hospital, Feigin Center, Houston, Texas, USA; 3 Department of Pediatrics, Center for Epidemiology and Population Health, Baylor College of Medicine, One Baylor Plaza, Houston, Texas, USA; 4 Department of Collective Health, Faculdade de Ciências Médicas da Santa Casa de São Paulo, São Paulo, Brazil; 5 Division of Epidemiology and Clinical Research, Department of Pediatrics, University of Minnesota, Minneapolis, Minnesota, USA; 6 Department of Pediatrics, University of Minnesota Masonic Cancer Center, Minneapolis, Minnesota, USA; 7 Department of Pediatrics, Neuroscience Institute, Children’s Minnesota, Minneapolis, Minnesota, USA; 8 Department of Mathematics and Statistics, University of New Mexico, Albuquerque, New Mexico, USA

**Keywords:** birth defects, childhood, epidemiology, Hodgkin lymphoma, risk factors

## Abstract

**Background:**

Non-chromosomal birth defects are an important risk factor for several childhood cancers. However, these associations are less clear for Hodgkin lymphoma (HL). Therefore, we sought to more fully elucidate the association between non-chromosomal birth defects and HL risk.

**Procedure:**

Information on cases (*n* = 517) diagnosed with HL (ages of 0–14) at Children’s Oncology Group Institutions for the period of 1989–2003 was obtained. Control children without a history of cancer (*n* = 784) were identified using random digit dialing and individually matched to cases on sex, race/ethnicity, age, and geographic location. Parents completed comprehensive interviews and answered questions including whether their child had been born with a non-chromosomal birth defect. To test the association between birth defects and HL risk, conditional logistic regression was applied to generate adjusted odds ratios (aORs) and 95% confidence intervals (CIs).

**Results:**

Children born with any non-chromosomal birth defect were not more likely to be diagnosed with HL at 0–14 years of age (aOR: 0.91; 95% CI: 0.69–1.21). No associations were detected between major or minor birth defects and HL (aOR: 1.34; 95% CI: 0.67–2.67 and aOR: 0.88; 95% CI: 0.57–1.34, respectively). Similarly, no association was observed for children born with any birth defect and EBV-positive HL (aOR: 0.57; 95% CI: 0.25–1.26).

**Conclusions:**

Previous assessments of HL in children with non-chromosomal birth defects have been limited. Using data from the largest case–control study of HL in those <15 years of age, we did not observe strong associations between being born with a birth defect and HL risk.

## 1 | INTRODUCTION

Hodgkin lymphoma (HL) is a lymphoid neoplasm arising from germinal or post-germinal center B-lymphocytes that have transformed into Hodgkin/Reed-Sternberg malignant cells, which often have a distinctive binucleate morphology in a background of various inflammatory cells.^1,2^ Incidence in children is approximately 5—10 cases per million per year, comprising 5–6% of all childhood cancers, with increasing incidence in older children and adolescents.^3^ In the United States, HL is the most common cancer among adolescents aged 15–19 years, representing 15% of all cancers diagnosed in that age group.^4^ While advancements in therapy have improved 5-year overall survival rates to over 94%, HL survivors often experience chronic health conditions due to their treatment or the original tumor.^5,6^ The long-term impact of these late effects underscores the importance of identifying novel risk factors and signifies a need for increased surveillance for childhood HL.

HL etiology is multifactorial, with diverse and complex factors implicated in pathogenesis.^7–9^ Epstein–Barr virus (EBV) infection and acute mononucleosis^10–12^ are associated with HL in approximately 40% of cases, more frequently in younger patients and in patients from some regions, such as sub-Saharan Africa, and positivity differs by subtype and is predominantly seen in mixed cellularity (MC) compared with nodular sclerosis (NS).^13,14^ Similarly, a history of other immune dysfunction phenotypes is associated with an increased risk of HL.^15–17^ While these have been explored, few established risk factors for HL exist, and with childhood HL understudied in general, surveillance and prevention strategies are limited.^18–25^

Birth defects are among the strongest risk factors for several childhood cancers.^26–30^ For example,^31–33^ children born with trisomy 21 have a 20- to 200-fold increased risk of developing acute lymphoblastic and myeloid leukemia, respectively.^34^ More recently, studies have also demonstrated that non-chromosomal birth defects (e.g., non-syndromic congenital heart disease) also increase the risk of childhood cancer.^27,30^ Birth defects are of interest in understanding childhood cancer etiology because they signal an error during embryonal or fetal development that may have genetic and/or epigenetic/environmental (teratogenic) underpinnings.^31–33^

While estimates of the association between birth defects and lymphoma have been reported as part of larger studies assessing birth defects and childhood cancer, most studies did not report results stratified by lymphoma subtype or EBV infection status,^27,29,30,35–41^ which are suspected as having differing etiologies.^42–44^ Additionally, the HL-specific estimates that have been reported are based on studies with small sample sizes.^45–47^ The relationship between birth defects and HL is an important question to examine because birth defects and HL might have overlapping or related etiologies. Birth defects are known to be caused by various genetic and environmental factors similar to those risk factors identified for HL, including monogenic and epigenetic regulation mechanisms, and biologic agents like viruses.^9,10,31,33,48^ An example to support the hypothesis that birth defects and HL may have similar etiologic origins is biliary atresia. Genetic variation in the HLA region is associated with biliary atresia risk.^49,50^ Genome-wide association and sequencing studies of HL have also confirmed that variation in the HLA region impacts HL risk,^7,51,52^ hence examining this question is warranted. Additionally, there has not been a study that focuses solely on assessing the potential relationship between birth defects and childhood HL. However, such a study is needed for at least two reasons: (1) fewer studies focus on risk factors specifically for childhood HL compared with HL that occurs in adolescents and young adults leaving the potential for yet-to-be-identified risk factors specific to this younger age group; and (2) birth defects often show stronger associations with cancer among younger children.^35,53^ Therefore, our objective was to evaluate this association by leveraging a Children’s Oncology Group (COG) study of incident childhood HL.

## 2 | METHODS

### 2.1 | | Study design and participant ascertainment

The current analysis utilized data originally collected in the early 2000s as part of the Children’s Cancer Group (CCG; now COG) Protocol E13: “Case–control study of Hodgkin’s Disease in children.” Details of this study have been previously described.^17,22^ Briefly, using the CCG/COG patient registry, cases were identified as eligible if they met the following criteria: (1) a pathologically confirmed diagnosis of HL between the ages of 0—14 years at participating CCG/COG institutions in the United States, Puerto Rico, or Canada between January 31, 1989 and July 28, 2003; (2) physician approval for contact; (3) telephone in residence; and (4) at least one biological parent who spoke English or Spanish and consented to participate in the interview. Deceased cases meeting the outlined criteria were also considered eligible for inclusion. After the treating physician granted permission to contact the family, an initial phone call was made to evaluate the interest of potential participants. All copies of study materials (study description, interview guide, and consent form) were sent to interested families by conventional mail.

Control children without a prior cancer diagnosis were identified by random digit dialing (RDD) and were individually matched to cases on the following attributes: area code as a proxy for geographic location, sex, race and ethnicity, and birth date (controls matched ±1 year for cases <5 years and ±3 years for cases 5—14 years). Potential control children were classified as eligible if they met the following criteria: (1) no history of cancer; (2) a telephone in residence; and (3) at least one biological parent who spoke English or Spanish and consented to participate in the interview. Since HL is rarely diagnosed in children between 0 and 9 years old, up to three controls were chosen per case in this age range to enhance statistical power. For cases diagnosed between the ages of 10—14 years, one control was selected. Control parents gave verbal authorization for participation and received the same study materials prior to the interview.

### 2.2 | Interviews

The E13 parent study used separate structured telephone interviews of up to two parents/guardians to gather participant demographic data and medical histories. Maternal interview responses were used preferentially in the current analysis. Families were contacted a mean of 1.11 (SD = 0.97) years after diagnosis for cases and 2.79 (SD = 1.97) years after the assigned reference date (case’s HL diagnosis date) for controls.

### 2.3 | Assessment and classification of birth defects

Detailed data on whether a child was born with a non-chromosomal birth defect were captured in the survey instrument. Parents were asked if the index children were born with: strawberry birthmark (hemangioma), extra fingers or toes, club foot, other limb deformities, cleft lip or palate, heart murmur, congenital heart defect (CHD) including but not limited to tetralogy of Fallot, ventricular septal defects and patent ductus arteriosus, cerebral palsy or any other specified congenital defects. Parents also provided information about the index child’s other medical and illness history. All birth defects reported by parents were subsequently coded using the International Classification of Diseases, Ninth Revision.^54^ Specifically, we evaluated structural birth defects with ICD-9 codes 740.0–759.9, consistent with previous assessments, as well as birth defects outside of this range (e.g., cystic fibrosis without meconium ileus ICD-9: 277; inguinal hernia ICD-9: 550.9) if reported by the parent.^55^ As our primary question was related to non-chromosomal birth defects, we excluded children with chromosomal anomalies or other genetic syndromes (*n*_cases_ = 2, *n*_controls_ = 5).

We assessed the presence or absence of a non-chromosomal birth defect in three ways: (1) being born with any defect, which included strawberry birthmarks (hemangioma), port wine stains, and other birthmarks (yes or no); (2) being born with any major birth defect versus no birth defect; and (3) being born with any minor birth defect versus no defect. If a participant had both major and minor birth defects, they were assessed as having a major birth defect and excluded from the estimates of minor birth defects. The differentiation between major and minor birth defects was based on previous assessments.^30,55^ The birth defects included in this study, and whether they were classified as major or minor, are indicated in Table S1.

### 2.4 | Clinical characteristics and tumor categorization

Clinical and pathologic data for all cases were collected from the treating CCG/COG institutions as part of the parent E13 study. Cases were categorized by HL histologic subtype: lymphocyte predominant (LP), MC, NS, or other subtypes. Archived formalin-fixed paraffin-embedded tumor specimens (*n* = 355) were obtained directly from the treating CCG/COG institutions. As part of the original E13 parent study, tumor EBV status was determined in these samples using a standard digoxigenin-based in situ hybridization technique by detecting EBV-encoded RNAs (EB virus-encoded small RNA [EBER]—1 and EBER-2).^11^ Positive controls included HL specimens known to be EBV-positive and B95-8 cells, while the negative controls were EBER sense probes. The original E13 parent study investigators confirmed that intact RNA had been preserved in all tumor specimens by detecting small nuclear RNA U6 using molecular probes.

### 2.5 | Statistical analysis

Distributions of selected parental and child demographic characteristic data obtained via the survey instrument for cases and controls were summarized (e.g., means and frequencies) by case status. *p* Values were generated for these comparisons by applying chi-squared tests for categorical variables or t-tests for continuous variables. Conditional logistic regression was used to maintain the individually matched case–control sets to evaluate the associations between being diagnosed with a birth defect and the risk of childhood HL. First, we generated unadjusted odds ratios (ORs) and 95% confidence intervals (CIs) to evaluate the association between maternal and perinatal characteristics and the occurrence of HL (i.e., univariate models). We next calculated adjusted ORs (aOR), including other independent variables if they were: (1) associated with the outcome in unadjusted analyses at *p* < .05 or (2) identified as risk predictors of HL in the literature. These covariates included maternal age at index child’s birth (reference = < 25 years of age), level of maternal education (reference = high school graduate), and annual household income (reference = $20,000–$39,999). We conducted these adjusted conditional logistic regression analyses overall, as well as stratified by: (1) EBV tumor positivity status (EBV^+^ or EBV^−^), (2) HL histologic subtype (NS, MC, LP), and (3) child’s age at HL diagnosis (0–4, 5—9, and 10—14 years). All analyses were conducted in Stata 16 (StataCorp LP).

### 2.6 | Protection of human subjects

The Institutional Review Boards at the original coordinating centers (University of Pittsburgh and the University of New Mexico), participating CCG/COG institutions, the University of Minnesota, and Baylor College of Medicine approved this study.

## 3 | RESULTS

Across the 117 participating CCG/COG North American institutions, 646 potentially eligible cases with childhood HL were identified for this study in the early 2000s. Interviews in the original E13 parent study were conducted in full for 517 of the cases (80%), of which there were 324 cases with NS (62.7%), 92 cases with MC (17.8%), 60 cases with LP HL (11.6%), 38 with other subtypes (7.4%), and three with missing subtype information (0.6%). To select controls, 207,438 telephone calls to 88,429 telephone numbers were selected via RDD sampling technique, and 1209 potential control matches to cases were identified. Of these, 784 (64.8%) were interviewed and incorporated in the original E13 parent study, 323 (26.7%) actively declined participation, 101 (8.4%) passively refused to participate, and one (0.1%) was ineligible.

Cases and controls were predominantly male (61.9 and 66.1%, respectively; Table 1). Less than 5% of cases were under the age of 5 years, increasing to 72.1% of cases between the ages of 10—14 years. Cases tended to have mothers who were younger at child’s birth compared with controls (*p* = .02), and also mothers with lower levels of educational attainment compared with control children (*p*=.001). Last, the difference in the distribution of household income differed by case status, where cases tended to be born in families with lower household incomes (*p* < .001). In this study population, those in the lowest income group were at the highest risk of developing HL in univariate analysis (OR: 1.45; 95% CI: 1.10–1.90). Markers of childhood socioeconomic status and affluence impact childhood HL risk differentially based on sub-type, with MC occurring more frequently in poorer socioeconomic environments and NS occurring at a higher incidence in more affluent environments.^14,56,57^

Of the 355 tumors (68% of case group) assayed for EBV-encoded RNAs, 84 had EBV RNAs detected (23.7% of tumors assayed), with the following distribution across age groups: five cases under 5 years of age (22.7%); 36 cases between the ages of 5—9 years (29.5%); and 43 cases 10 years and above (11.5%; Table 2). In contrast, 271 tumors did not have EBV RNAs detected (52.4%).

After excluding those individuals born with a chromosomal abnormality, 515 cases with HL and 779 controls were included in the association testing. In this cohort, 151 birth defects were reported among 136 cases with HL (26.4%), and 222 birth defects were reported among 201 controls (25.8%; Table 3), with these counts reflecting multiple birth defects per participant if applicable (Table S1). When considering being born with any defect, not reflecting multiple birth defects, 136 cases (26.4%) and 201 controls (25.8%; Table 3) were reported to have been diagnosed with any birth defect. When considering those born with any major defect, 36 cases (7.0%) and 57 controls (7.3%) reported having any major birth defect. One hundred cases (19.4%) and 144 controls (18.5%) were reported to have been diagnosed with a minor birth defect.

Neither the presence of any birth defect nor any major birth defect was associated with HL (aOR: 0.91; 95% CI: 0.69–1.21, and aOR: 0.84; 95% CI: 0.50–1.40, respectively; Table 3), nor with any of the subgroups examined. As with HL overall, associations were not detected between being born with any birth defect and either EBV-positive or EBV-negative HL (aOR: 0.57; 95% CI: 0.25–1.26, and aOR: 0.98; 95% CI: 0.68–1.44, respectively).

There were also no associations detected when we examined birth defects and specific HL subtypes (Table 4). For example, children born with any birth defect were neither more nor less likely to develop NS HL (aOR: 0.99; 95% CI: 0.69–1.43). No strong associations were detected when evaluating associations by age at diagnosis (Table 5). Being born with any defect was not associated with an HL diagnosis between the ages of 5—9 years (aOR: 0.60; 95% CI: 0.32–1.12). Likewise, being born with a major birth defect was not found to impact risk of an HL diagnosis between the ages of 10—14 years (aOR: 1.85; 95% CI: 0.86–3.98).

## 4 | DISCUSSION

In this case–control study of HL with data collected in the early 2000s, we did not observe associations between non-chromosomal birth defects and HL. A clear relationship between having a birth defect and developing a specific HL histologic subtype was not evident. Results from previous studies evaluating birth defects and lymphoma risk were largely equivocal. Some studies suggested that children born with either chromosomal or non-chromosomal birth defects are at increased risk of developing any lymphoma. For example, Rankin et al.^38^ reported that children with birth defects had a fivefold increased risk of developing any lymphoma (risk ratio [RR]: 5.3; 95% CI: 2.4–12.0; *n* = 66); Carozza et al.^29^ reported a 1.8-fold increased risk (incidence rate ratio [IRR]: 1.8; 95% CI: 0.8–3.6; *n* = 145); Fisher et al.^27^ reported a 2.2-fold increased risk in children with non-chromosomal birth defects (hazard ratio [HR]: 2.2; 95% CI: 1.4–3.6; *n* = 422); and Janitz et al.^35^ described that children with non-chromosomal defects had a threefold increased risk (HR: 3.0; 95% CI:1.1–8.4; *n* = 40). However, other studies suggested a lack of positive associations or even inverse associations. For example, Agha et al.^41^ found that children who were born with a birth defect were not more or less likely to develop lymphoma compared with children born without birth defects (IRR: 1.0; 95% CI: 0.6–1.6; *n* = 17). In a Swedish population-based cohort study of CHDs, after adjustment for Down syndrome, CHD was associated with increased risk of lymphoma (HR: 1.6; 95% CI: 1.1– 2.4; *n*=1,273). In this cohort, an elevated risk of lymphoma (HR=8.13, 95% CI: 4.06–16.30) was also observed in children with complex CHD,^58^ as previously reported.^59,60^ While this was not observed in our study, it could be due to the limited number of cases (*n* = 4) and controls (*n* = 7) with a CHD reported in this study population (Table S1). Additionally, the proportion of children with a birth defect, the range of included defects, and the source of information on defects differ between the current study and those previously published, which could help explain differences in results across studies. It is critical to highlight that most of these studies did not evaluate HL independently. Those studies evaluating the association between birth defects and HL have been based on smaller case sizes than that presented here (*n* = 517), such as: Schraw et al.^46^ in which 345 cases with HL were evaluated (*q*-value: .12); Schumacher et al.^61^ in which 54 cases with HL were assessed (non-significant [NS] at *p* = .05); Mann et al.^47^ in which 32 cases with HL were evaluated (NS at *p* = .05); and Botto et al.^45^ in which fewer than 10 cases with HL were reported on (IRR: 1.0; 95% CI: 0.1–9.2).

There are several reasons why non-chromosomal birth defects may not be associated with HL compared with other childhood cancers. First, the etiologic origins of all childhood cancers are not uniform. In fact, HL appears to arise from immune dysregulation or impaired immune function in some cases.^15,17,62^ Thus, development processes that lead to structural birth defects may not be as crucial for HL risk, or birth defects associated with syndromes, including immune dysfunction (e.g., Fanconi anemia), could represent a minor contribution to HL pediatric population. Second, the age of onset for HL is typically later than for other lymphomas and many childhood cancers, suggesting the mechanisms that give rise to non-chromosomal birth defects may not play as important an etiologic role in HL development. This is supported by previous studies suggesting cancer risk is strongest among younger individuals.^46,53^

This study should be considered in light of certain limitations. First, we relied on maternal report to identify birth defects rather than on registry-based or clinical assessments. This could limit our ability to evaluate certain birth defects (e.g., minor birth defects). However, other assessments relying on maternal report^63^ have been consistent with registry-based linkage studies^26^ and clinical examinations.^64^ Based on this, we think this would not adversely impact our assessment. We do note that a higher prevalence of birth defects among cases and controls was reported in the current study compared with population-based assessments. We might expect parents of children with HL to be hypervigilant about recalling any birth defects in their children, as they often try to figure out what caused their child’s cancer. However, we might expect this to cause spurious associations, as we would not expect the control parents to recall birth defects equally well. However, in these data, both case and control parents report birth defects, particularly minor birth defects, in nearly equal proportions. Second, due to limited sample size, we could not test the associations between specific birth defects (e.g., cleft palate) and HL risk, and multiple birth defects and HL risk. Third, we were restricted to specific HL subtype analyses due to limited sample size and could not report estimates for those ages less than 5 years due to sparse data. Cases also tended to have mothers who were younger at child’s birth compared with controls (*p* = .02), which is indicative of selection bias, something that is seen in other retrospective interview-based case–control studies of childhood cancer.^17,22^ For example, older mothers would be expected to have childrenwith more birth defects. To address this limitation, we therefore controlled for maternal age at index child’s birth in our analyses. Despite these limitations, our study has notable strengths. This study was based on a large case group (*n* = 517)^17,22^: more HL cases than reported on in previous studies, as outlined above. Further, prior evaluations reported unadjusted effect estimates, while herein, we report effect estimates adjusted for potential confounding factors. The size of our study population allowed us to conduct subgroup analyses by HL subtype, tumor EBV status, and age for categories with adequate sample sizes. In this study, we also considered birth defects as either major defects or aminor defects, something not previously reported in relation to HL.

In summary, our assessment does not demonstrate that nonchromosomal birth defects are important risk factors for childhood HL. Based on this, we recommend that future studies evaluating these relationships focus on other malignancies with an earlier mean age of onset and arising in more immature cell types (e.g., neuroblastoma).^26^ Additionally, novel assessments and approaches are needed to identify novel risk factors for developing HL.

## Supplementary Material

Supplemental Table 1 downloaded from PBC

## Figures and Tables

**TABLE 1:** Selected maternal and participant characteristics of 517 childhood and adolescent Hodgkin lymphoma cases and 784 matched controls.

Characteristic	Cases (*n* = 517)	Controls (*n* = 784)	OR^a^ (95% CI)	*p* Value
Child sex, *n* (%)				–
Male	320 (61.9)	518 (66.1)	–	
Female	197 (38.1)	266 (33.9)		

Child age (years), *n* (%)				–
0–4	22 (4.3)	97 (12.4)	–	
5–9	122 (23.6)	302 (38.5)		
10–14	373 (72.1)	326 (41.6)		
15+	0 (0)	59 (7.5)		

Child race/ethnicity, *n* (%)				–
Non-Hispanic White	386 (74.7)	631 (80.5)	–	
Hispanic	62 (12.0)	78 (9.9)		
Non-Hispanic Black	54 (10.4)	67 (8.5)		
Non-Hispanic Asian/Pacific Islander	6 (1.2)	2 (0.3)		
Other	9 (1.7)	6 (0.8)		

Birth weight (grams), *n* (%)				.91
≤2499	33 (6.4)	45 (5.7)	1.05 (0.66–1.67)	
2500–3999	401 (77.6)	616 (78.6)	Reference (1.00)	
≥4000	80 (15.5)	117 (14.9)	1.16 (0.83–1.61)	
Unknown	3 (0.6)	6 (0.8)		
Mean (SD)	3438.8 (588.1)	3420.6 (560.2)		.57

Birth order, *n* (%)				.37
1st	208 (40.2)	309 (39.4)	Reference (1.00)	
2nd	163 (31.5)	271 (34.6)	0.93 (0.71–1.21)	
≥3rd	140 (27.1)	189 (24.1)	1.20 (0.90–1.61)	
Unknown	6 (1.2)	15 (1.9)		

Number of siblings, *n* (%)				.07
0	30 (5.8)	68 (8.7)	0.77 (0.48–1.24)	
1	192 (37.1)	296 (37.8)	Reference (1.00)	
2	147 (28.4)	237 (30.2)	0.93 (0.70–1.25)	
3+	148 (28.6)	183 (23.3)	1.20 (0.88–1.62)	

Maternal age, *n* (%)				.02
≤25	248 (48.0)	315 (40.2)	Reference (1.00)	
25–29.99	164 (31.7)	272 (34.7)	0.80 (0.61–1.04)	
≥30	104 (20.1)	195 (24.9)	0.73 (0.54–1.00)	
Unknown	1 (0.2)	2 (0.3)		

Maternal education, *n* (%)				.001
Less than HS	73 (14.1)	65 (8.3)	1.82 (1.20–2.76)	
12 years/GED/HS graduate	175 (33.8)	248 (31.6)	Reference (1.00)	
Post HS training/some college	182 (35.2)	282 (35.9)	0.91 (0.69–1.22)	
College graduate and above	85 (16.4)	186 (23.7)	0.61 (0.44–0.86)	
Unknown	2 (0.39)	3 (0.38)		

Maternal prenatal vitamin use, *n* (%)				.51
Used	442 (85.5)	687 (87.7)	Reference (1.00)	
Not used	69 (13.3)	96 (12.2)	1.09 (0.77–1.54)	
Unknown	6 (1.2)	1 (0.1)		

Household income, *n* (%)				<.001
$0–$19,999	243 (47.0)	268 (34.2)	1.45 (1.10–1.90)	
$20,000–$39,999	206 (39.8)	333 (42.5)	Reference (1.00)	
$40,000	53 (10.2)	152 (19.4)	0.59 (0.40–0.88)	
Unknown	15 (2.9)	31 (3.9)		

a OR is unadjusted; CIs, confidence intervals; *n*, number of cases or controls; ORs, odds ratios; –, ORs, 95% CIs and *p* values not calculated for matching factors: child’s sex, age, and race/ethnicity.

**TABLE 2:** Distribution of sex, tumor EBV status, and histologic subtype for 517 childhood and adolescent Hodgkin lymphoma cases by age group.

Characteristic	Overall (*n* = 517)	0–4 years (*n* = 22)	5–9 years (*n* = 122)	10–14 years (*n* = 373)
Child sex, *n* (%)				
Male	320 (61.9)	20 (90.9)	88 (72.1)	212 (56.8)
Female	197 (38.1)	2 (9.1)	34 (27.9)	161 (43.2)

EBV tumor status, *n* (%)				
EBV+	84 (16.2)	5 (22.7)	36 (29.5)	43 (11.5)
EBV−	271 (52.4)	10 (45.5)	48 (39.3)	213 (57.1)
EBV undetermined	162 (31.4)	7 (31.8)	38 (31.1)	117 (31.4)

HL subtype, *n* (%)				
Nodular sclerosis	324 (62.7)	7 (31.8)	48 (39.3)	269 (72.1)
Mixed cellularity	92 (17.8)	10 (45.5)	35 (28.7)	47 (12.6)
Lymphocyte predominant	60 (11.6)	3 (13.6)	24 (19.7)	33 (8.8)
Other	38 (7.4)	1 (4.5)	15 (12.3)	22 (5.9)
Subtype missing	3 (0.6)	1 (4.5)	0 (0.0)	2 (0.5)

**TABLE 3:** Association between birth defects and childhood and adolescent Hodgkin lymphoma, overall and by EBV status.

	Combined cases			EBV+			EBV−		
	Cases (*n*)	Controls (*n*)	aOR^a,b^ (95% CI)	Cases (*n*)	Controls (*n*)	aOR^a,b^ (95% CI)	Cases (*n*)	Controls (*n*)	aOR^a,b^ (95% CI)
Any birth defect									
No	379	578	Reference (1.00)	65	121	Reference (1.00)	186	274	Reference (1.00)
Yes	136	201	0.91 (0.69–1.21)	19	40	0.57 (0.25–1.26)	84	105	0.98 (0.68–1.44)

Any major defect									
No	379	578	Reference (1.00)	65	121	Reference (1.00)	186	274	Reference (1.00)
Yes	36	57	0.84 (0.50–1.40)	4	12	—	24	28	1.02 (0.52–1.99)

Any minor defect									
No	379	578	Reference (1.00)	65	121	Reference (1.00)	186	274	Reference (1.00)
Yes	100	144	0.93 (0.66–1.29)	15	28	0.70 (0.28–1.75)	60	77	0.91 (0.58–1.41)

a Cases and controls matched on the following factors: child’s sex, age, race/ethnicity, and geographic location.

b aOR adjusted for maternal education, maternal age, and household income; –, adjusted estimate not shown due to cell counts less than five; aOR, adjusted odds ratios; CIs, confidence intervals; *n*, number of cases or controls.

**TABLE 4:** Association between birth defects and childhood and adolescent Hodgkin lymphoma by subtype.

	Nodular sclerosis			Mixed cellularity			Lymphocyte Predominant		
	Cases (*n*)	Controls (*n*)	aOR^a,b^ (95% CI)	Cases (*n*)	Controls (*n*)	aOR^a,b^ (95% CI)	Cases (*n*)	Controls (*n*)	aOR^a,b^ (95% CI)
Any birth defect									
No	233	318	Reference (1.00)	71	125	Reference (1.00)	45	78	Reference (1.00)
Yes	91	106	0.99 (0.69–1.43)	20	48	0.58 (0.27–1.24)	14	31	0.78 (0.34–1.76)

Any major defect									
No	233	318	Reference (1.00)	71	125	Reference (1.00)	45	78	Reference (1.00)
Yes	26	27	1.34 (0.67–2.67)	3	15	–	4	9	–

Any minor defect									
No	233	318	Reference (1.00)	71	125	Reference (1.00)	45	78	Reference (1.00)
Yes	65	79	0.88 (0.57–1.34)	17	33	0.80 (0.33–1.92)	10	22	0.90 (0.34–2.38)

a Cases and controls matched on the following factors: child’s sex, age and race/ethnicity, and geographic location.

b aOR adjusted for maternal education, maternal age, and household income; –, adjusted estimate not shown due to cell counts less than five; aOR, adjusted odds ratios; CIs, confidence intervals; *n*, number of cases or controls.

**TABLE 5:** Association between birth defects and childhood and adolescent Hodgkin lymphoma by age group.

	0–4			5–9			10–14		
	Cases (*n*)	Controls (*n*)	aOR^a,b^ (95% CI)	Cases (*n*)	Controls (*n*)	aOR^a,b^ (95% CI)	Cases (*n*)	Controls (*n*)	aOR^a,b^ (95% CI)
Any birth defect									
No	20	74	Reference (1.00)	102	221	Reference (1.00)	257	241	Reference (1.00)
Yes	2	23	–	20	80	0.60 (0.32–1.12)	114	83	1.17 (0.77–1.76)

Any major defect									
No	20	74	Reference (1.00)	102	221	Reference (1.00)	257	241	Reference (1.00)
Yes	0	5	–	4	24	–	32	22	1.85 (0.86–3.98)

Any minor defect									
No	20	74	Reference (1.00)	102	221	Reference (1.00)	257	241	Reference (1.00)
Yes	2	18	–	16	56	0.62 (0.31–1.24)	82	61	0.95 (0.58–1.57)

a Cases and controls matched on the following factors: child’s sex, age and race/ethnicity, and geographic location.

b aOR adjusted for maternal education, maternal age, and household income; –, adjusted estimate not shown due to cell counts less than five; aOR, adjusted odds ratios; CIs, confidence intervals; *n*, number of cases or controls.

## Abbreviations:

aOR: adjusted odds ratio
CCG: Children’s Cancer Group
CHDs: congenital heart defects
CI: confidence interval
COG: Children’s Oncology Group
E13: Protocol E13: “Case–control study of Hodgkin’s Disease in children.”
EBER: Epstein–Barr virus-encoded small RNA
EBV: Epstein–Barr Virus
HL: Hodgkin lymphoma
HR: hazard ratio
ICD-9: International Classification of Diseases, Ninth Revision
IRR: incidence risk ratio
LP: lymphocyte predominant
MC: mixed cellularity
NS: nodular sclerosis
NS: nonsignificant
OR: odds ratio
P: *p* value
RDD: random digit dialing
RR: risk ratio
SD: standard deviation
